# Sound-Induced Flash Illusions Support Cortex Hyperexcitability in Fibromyalgia

**DOI:** 10.1155/2022/7355102

**Published:** 2022-09-25

**Authors:** Vincenzo Di Stefano, Salvatore Iacono, Andrea Gagliardo, Bruna Maggio, Giuliana Guggino, Massimo Gangitano, Roberto Monastero, Vito Renato Maggio, Nadia Bolognini, Filippo Brighina

**Affiliations:** ^1^Section of Neurology, Department of Biomedicine, Neuroscience and Advanced Diagnostics (BIND), University of Palermo, Palermo 90127, Italy; ^2^Department of Health Promotion, Mother and Child Care,Internal Medicine and Medical Specialties (DIBIMIS), University of Palermo, Palermo 90127, Italy; ^3^National Social Insurance Agency (INPS), Agrigento 92100, Italy; ^4^Department of Psychology, Milan Center for Neuroscience–NeuroMi, University of Milano Bicocca, Milano 20126, Italy; ^5^Laboratory of Neuropsychology, IRCCS Istituto Auxologico Italiano, Milan, Italy

## Abstract

**Objectives:**

Fibromyalgia (FM) is characterized by spontaneous chronic widespread pain in combination with hyperalgesia to pressure stimuli. Sound-induced flash illusions (SIFIs) reflect cross-modal interactions between senses allowing to assess a visual cortical hoerexcitability (VCH) by evaluating the fission and fusion illusions disruption. The aims of the present study were to explore whether SIFIs are perceived differently in patients with fibromyalgia as compared to healthy controls (HCs) and how migraine affects fission and fusion illusions in fibromyalgia.

**Methods:**

A single flash (F) accompanied by 0 to 4 beeps (B) was presented to induce the fission illusion while multiple flash (i.e., 2 to 4) accompanied by 0 or 1 beep was presented to induce fusion illusion. The mean number of perceived flashes in fission and fusion illusion trials was compared between the groups (i.e., FM, FM with migraine, and HCs) using repeated-measures analysis of variance. Medication history was recorded along with the administration of Fibromyalgia Impact Questionnaire and Hospital Anxiety and Depression scales.

**Results:**

Twenty-four patients with FM (mean age 51, 2 ± 10, 6 years; 22 females), seventeen patients with FM and migraine without aura (mean age 47.8 ± 11.4 years; 16 females; 13 chronic, 4 episodic migraine), and forty-one age- and sex-matched HCs (mean age 47.3 ± 6.9 years; 34 females) participated in the study. Fission and fusion illusory effects were detected in all the participants. However, in FM patients, the fission illusion was reduced and almost abolished as compared to HCs (1F1B, *p* = 0.02; 1F2B, *p* < 0.0001; 1F3B, *p* < 0.0001; 1F4B, *p* = 0.0001), while there were no differences between groups in fusion trials. Migraine did not affect the fission and the fusion illusions.

**Conclusion:**

Results from this study confirm that patients with FM have a VCH suggesting that the pathological changes in cortical excitability might have important roles in the pathophysiology of FM. SIFI represents a noninvasive behavioral tool for the exploration of cross-sensory functional interplay.

## 1. Introduction

Sound-induced flash illusions (SIFIs) are a clear example of cross-modal interactions between senses [1, 2]: a single flash is perceived as multiple flashes if paired with multiple beeps (i.e., fission illusion); conversely, fewer flashes are perceived than those actually presented when coupled with a single beep (i.e., fusion illusion) [1]. The perception of SIFIs is associated to the level of excitability of visual and auditory cortices. For instance, in healthy subjects, the fission illusion can be disrupted by increasing the excitability of the primary visual cortex with anodal transcranial direct current stimulation (tDCS) or by decreasing the excitability of the superior temporal cortex with cathodal tDCS [3]. In a recent study, we have taken advantage of the SIFIs to explore the level of cortical excitability in patients affected by migraine given the well-known prominent role of visual cortical hyperexcitability (VCH) in its pathophysiology [4]. The SIFIs allowed us to evaluate how the abnormal cortical excitability affects the perception of these cross-modal illusions, hence confirming the influence of VCH on multisensory interactions in migraine. So far, apart from migraine, there are no studies exploring VCH in other painful conditions. Fibromyalgia (FM) is characterized by spontaneous chronic widespread pain in combination with hyperalgesia to pressure stimuli [3, 4]. In patients with FM, a state of central hyperexcitability of the nociceptive system leads to exaggerated pain after sensory stimulation due to an increased perception of pain stimuli [6–8]; this seems to be explained by spinal cord mechanisms [6] and C nociceptor dysfunction [9]. Recent evidence supports a combination of C-fiber hyperexcitability [10], as well as loss of A-delta fiber integrity, in patients with FM [10–14], which may contribute to the imbalance of excitatory and inhibitory neurotransmission. Of interest, recent evidence supports a role for brain hyperexcitability in the pathophysiology of fibromyalgia: an imbalance between excitatory and inhibitory neurotransmission in the insula has been linked to increased pain sensitivity in FM [15]. Furthermore, neurostimulation of brain areas involved in pain processing and control was shown to affect cognitive and affective FM symptoms [16]. In the present study, we explored whether the perception of SIFIs is altered in patients affected by FM to evaluate whether an abnormal cortical excitability might affect cross-modal processing. The impact of concurrent migraine in SIFIs perception was also evaluated.

## 2. Materials and Methods

We performed a case-control study with 1 : 1 case/control ratio including forty-one subjects attending to Neurology Unit of the University Hospital “P. Giaccone” of Palermo, Italy, fulfilling the following inclusion criteria: age greater or equal to 18 years and diagnosis of fibromyalgia according to diagnostic criteria [17]. The exclusion criteria included: age less than 18 years, diagnosis of epilepsy, hearing loss, ocular blindness, and absence/denied informed consent. The presence of migraine and the use of medications were not considered as exclusion criteria; therefore, migraine and medication histories were collected. The Fibromyalgia Impact Questionnaire (FIQ) [18] and the Hospital Anxiety and Depression Scale (HADS) [19] were administered to recruited patients.

Forty-one healthy controls (HCs), age- and sex-matched with patients, were recruited among patients' partners and caregivers (Table 1).

All participants underwent SIFIs: to assess fission illusory effects, a single flash (F) paired with multiple beeps (B) (i.e., 1F0B, 1F1B, 1F2B, 1F3B, 1F4B), while fusion effects were assessed by presenting multiple flashes with a single beep (i.e., 2F0B, 2F1B, 3F0B, 3F1B, 4F0B, 4F1B). Five fission trials and 6 fusion trials were presented for each fission and fusion condition. All the participants were tested in a dimly illuminated room at approximately 57 cm from a CRT computer monitor (Samsung SyncMaster 1200NF: resolution 1.024 ± 768, refresh rate 75 Hz) with their eyes aligned to the center of the screen and their head supported by a chinrest. Each trial began with the appearance of a white fixation cross, displayed at the center of the black screen (luminance = 0.02 candela [cd]/m^2^). At the eccentricity of 5° of the visual field, a white disk subtending at 2° was flashed 1–4 times. Two speakers, delivering the auditory stimulus, were positioned near the screen, and aligned with the flashes. Each flash (luminance = 118 cd/m2) and beep (intensity = 80 dB sound pressure level) lasted once the screen was refreshed (13 ms). The first flash appeared 26 ms after the first beep. We used a stimulus onset asynchrony of 65 ms (5 refreshes) between flashes and 52 ms (4 refreshes) between beeps. Eight trials were presented in random order for each experimental condition (total number of trials = 88; task duration approximately 5 minutes). For each trial, we instructed participants to report the number of perceived flashes. Before the experiment, we presented 10 practice trials, which were not considered in the analyses.

Quantitative variables were reported as means with standard deviation and they were compared by using one-way ANOVA. Qualitative variables were expressed as percentages, and then compared using the Chi-square test. To assess the presence of the fission illusory effects we analyzed participants' mean number of perceived flashes for every flash trial (1 flash combined with 0 to 4 beep) using repeated-measures analysis of variance (rmANOVA), with the between-subjects factor (2 levels: HCs and patients with FM) and the within-subjects factors beep (5 levels: 1F0B, 1F1B, 1F2B, 1F3B, 1F4B). To assess the fusion illusory effect, the participants' mean of perceived flash for every flash trial (from 1 to 4 flashes combined with 0 or 1 beep) was analyzed through rmANOVA with the between-subjects factor (group) and the within-subject factor beep (2 levels: *B* = 0–1). The relationship between clinical (i.e., FIQ, HADS-A, and HADS-D) and neurophysiological scores (i.e., the mean number of flashes perceived) was studied by using Spearman' correlation. The statistical analyses were performed using SPSS software (version 26.0, IBM Statistics, IBM Corp); the level of significance was set at *p* value < 0.05. The study was approved by the Ethical Committee ‘‘Palermo 1” of the Policlinico Paolo Giaccone, University of Palermo (*n*°5/2021).

## 3. Results

Forty-one patients affected by FM and 41 HCs completed the study (Table 1). Among patients with FM, the 41% (*n* = 17) were also affected by migraine with greater prevalence of chronic migraine (76.5%) than episodic ones (23.5%). The FIQ, HADS-A, and HADS-D scores in patients with FM were higher according to the presence of migraine but this was not statistically significant (Table 1). Thirteen FM patients with migraine (71%) and seventeen patients with FM without migraine (76%) were taking some medications at the time of the study, without difference between the groups (*p*=0.69). Medications were almost antidepressants (27%; i.e., duloxetine, venlafaxine, and amitriptyline) followed by antiepileptic drugs (7%; i.e., topiramate), analgesic (13%; i.e., nonsteroidal anti-inflammatory drugs, tramadol), vitamins (23%; i.e., vitamin *D*, B12, B6), or their association (30%) (Table 1).

### 3.1. Fission Illusions

**Figure 1:**
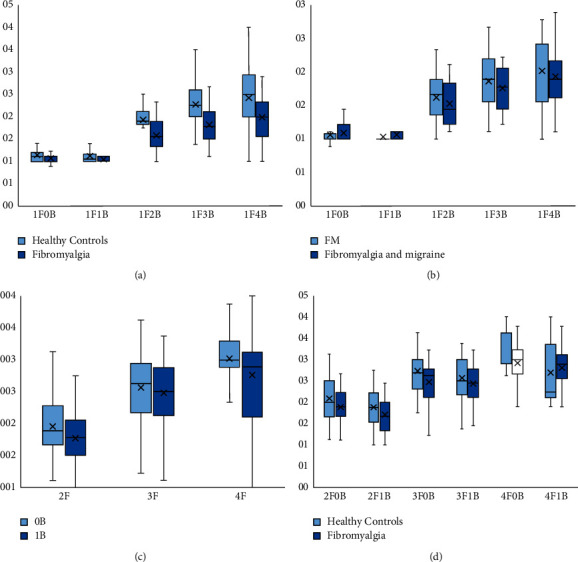
Box plots of SIFI for fission illusion (a–b) and fusion illusion trials (c–d). Fission illusion trials in patients affected by fibromyalgia compared to healthy controls (a) and comparison of fission illusion trials in patients with fibromyalgia with and without migraine (b). Comparison between 0B and 1B trials showing fusion illusory effect in all participants (c). Fusion illusions in patients affected by fibromyalgia compared to healthy controls (d). The fission and fusion illusion trials and the mean number of perceived flashes are reported in the horizontal and vertical axes, respectively. Median and interquartile ranges are reported.

The fission illusory effect was lower in patients affected by FM independently of the presence of migraine, as compared to healthy controls. Indeed, patients with FM reported overall fewer flashes than healthy controls in fission illusion trials (*p* < 0.001; Figure 1(a). Although for the 1F0B trial there were no significant differences (F_1F0B_ = 2.62, *p*=0.11), for 1F1B trial (F_1F1B_ = 5.74, *p*=0.02) and every other illusory trial (F_1F2B_ = 22.79, *p* < 0.001; F_1F3B_ = 22.45, *p* < 0.001; F_1F4B_ = 11.23, *p*=0.001) the fission illusory effect was marked (Table 2). The presence of migraine in FM showed a little summative effect in reducing the mean number of perceived flashes in some fission experimental conditions although this effect did not reach the significance level (F_1F0B_ = 0.59, *p*=0.51, F_1F1B_ = 0.28, *p*=0.27, F_1F2B_ = 0.42, *p*=0.39; F_1F3B_ = 0.10, *p*=0.4; F_1F4B_ = 0.18, *p*=0.7; Figure 1(b); Table 3. There were no significant correlations between the mean number of perceived flashes in fission illusion trials and FIQ, HADS-A, and HADS-D scores. The overall fission illusion trials were not different in FM with or without migraine depending on the type of medication (overall *p* > 0.05).

### 3.2. Fusion Illusions

Only 24 healthy controls (58%, 3 males and 21 females) completed the fusion illusion trials, whereas none of the patients with FM withdrew from the tasks. The fusion illusory effect was shown in all participants as they perceived fewer flashes when 1 beep was presented compared to 0-beep trials (2F: *p* < 0.0001; 3F: *p* < 0.02; 4F: *p* < 0.0001; Figure 1(c); Table 4). The mean number of reported flashes in overall 1 B trials were similar between healthy controls and patients with FM without difference between groups in 2F1B (*p*=0.7), 3F1B (0.3), and 4F1B (*p*=0.49) trials Figure 1(d); Table 5. There were no significant differences depending on the presence of migraine as well as there were no correlations between clinical scores and fusion illusion trials. The overall fusion illusion trials were not different in FM with or without migraine depending on the type of medication (overall *p* > 0.05).

## 4. Discussion

In this study, the difference in illusory cross-modal perceptions between patients affected by FM with and without migraine and healthy controls were investigated to evaluate the presence of VCH in patients with FM. Also, the role of migraine in FM was investigated. In a recent study, we have shown that migraineurs are generally less prone to perceive illusory cross-modal fission phenomena compared with controls [4]. We showed that the fission illusion was reduced in patients with FM compared to healthy controls (Figure 1(a)), whereas there were no differences with respect to the fusion illusion (Figure 1(d)). However, there was no significant effect of migraine on cross-modal perception in FM (Figure 1(b)). Therefore, our results support a state of hyperexcitability of the visual cortex in patients affected by FM in a nonmigraine dependent manner. In our study, the 41% of FM patients suffered from migraine (chronic migraine 76.5%) supporting previous evidence of a closer relationship between these two diseases [20] that would allow us to suspect that FM and chronic migraine may share similar pathophysiological mechanisms. Moreover, FM was more common in female migraineurs and in chronic migraine as well as in patients with aura symptoms; also, FIQ score positively correlated with headache frequency and disability scores such as Migraine Disability Assessment Scale and Headache Impact Test [21]. Furthermore, patients with both FM and migraine were found to have more depressive symptoms [22]. In our population, chronic migraine was more common in females with FM; also, patients with both FM and migraine showed higher FIQ, HADS-D, and HADS-A scores compared to patients with only FM, although, these differences were not statistically significant. However, these data could suggest that the association between chronic migraine and FM can worsen the patients' quality of life. Based on our results, patients with FM might have a generalized increased cortical excitability as migraineurs, which can be easily assessed with a simple behavioral test such as that of SIFIs. Indeed, recent studies suggest a role for brain hyperexcitability in the pathophysiology of fibromyalgia. Data from functional MRI showed a higher and more widespread BOLD signal response in frontal regions in fibromyalgia compared to health subjects during pain onset [23]; this brain hyperactivation may reflect the generalized hypervigilance to salient stimuli in fibromyalgia [24]. Magnetic resonance spectroscopy showed an imbalance between glutamic and GABAergic neurotransmission in the patients affected by FM [15]. Furthermore, neurostimulation protocols have been explored in FM with effects on cognitive and affective symptoms; of note, TMS studies showed an increased resting motor threshold and decreased MEPs amplitude in fibromyalgia [16]. This might suggest a lower threshold for activating the homeostatic mechanisms for the downregulation of cortical excitability, representing a compensatory phenomenon to avoid potentially dangerous increases in cortical activation. Moreover, cognitive dysfunctions have been related to reduced frontal activity and altered modulation of attention during complex tasks in FM [25]; EEG studies confirm the increased beta band connectivity [26] and reduced alpha-2 power spectrum in central, temporoparietal, and occipital brain areas [27] in the resting-state EEG; however, no definitive conclusion has yet been reached about the role of cortical excitability in the pathogenesis of fibromyalgia. Additionally, an autonomic dysfunction in FM has been hypothesized, but it was not confirmed in a recent study [24]. In our opinion, reduced illusory fission effects in FM might indicate an increased visual cortical responsivity, supporting the potential role of a condition of generalized cortical hyperexcitability.

In contrast to the fission illusion, patients affected by FM and HCs similarly perceived the fusion illusion. We hypothesize that the fusion illusion is less dependent on the level of VCH, hence, it is less prone to be influenced by its changes. This is in agreement with data from a recent report showing that tDCS modulates the fission, but not the fusion and illusion [3].

Cross-modal illusions, given their simplicity and reliability, should be explored in other diseases putatively linked to an abnormal level of cortical excitability. Moreover, FM provides a model for the study of how abnormal cortical excitability affects multisensory processing. In our perspective, hyperresponsivity to sensory stimuli, induced by disproportionate cortical hyperexcitability, may prevent optimal audio-visual interactions, making patients affected by fibromyalgia less vulnerable to cross-modal illusions.

There are some methodological limitations of our study that need to be considered. First, because of technical issues, 17 healthy controls did not complete the fusion illusion trials, consequently limiting their statistical analysis. Moreover, we evaluated SIFI in a single session apart from the severity of pain and symptoms of patients; we are aware that pain intensity can vary significantly throughout the daytime in fibromyalgia, and this might have in part biased the results obtained. Moreover, although patients affected by FM or migraine had a chronic disease condition characterized by VCH, for the study design, we are not able to distinguish if these alterations are primitive or secondary to a condition of chronic pain. Moreover, we did not report data on follow-up on SIFIs, which could be interesting to see how hyperexcitability develops as fibromyalgia progresses. Finally, medications were not discontinued because of chronic pain in FM or migraine may be highly disabling; hence, the interruption of therapy may result in unnecessary suffering. However, this could only represent a minor limitation since fission and fusion illusion trials in patients were not affected by medications. Hence, further studies are needed to clarify the role of cortical hyperexcitability in FM and whether cortex excitability comes early or late in the clinical course of FM.

## 5. Conclusions

Results from this study confirm that patients with fibromyalgia have a VCH which might be an epiphenomenon of generalized cortical hyperexcitability. Moreover, SIFIs represent a valid and simple tool for the exploration of cross-sensory interactions under conditions of VCH. Future studies are needed to better explore the well-known association between migraine and fibromyalgia.

## Figures and Tables

**Table 1:** Demographic and clinical features of the study population. FIQ, fibromyalgia impact questionnaire; HADS-A and HADS-D, Hospital Anxiety and Depression Scale. Variables are expressed as mean with standard deviation (SD) and numbers/count percentage.

	FM (*n* = 24)	FM with migraine (*n* = 17)	Healthy controls (*n* = 41)	*p*
Age (mean ± SD)	51.2 ± 10.6	47.8 ± 11.4	47.3 ± 6.9	0.23
Gender (female)	22 (92%)	16 (94%)	34 (83%)	0.4
FIQ (mean ± SD)	68.5 ± 10.4	71.1 ± 12.7		0.47
HADS-A (mean ± SD)	12.8 ± 4,1	12.8 ± 3,7		0.98
HADS-D (mean ± SD)	9.6 ± 3.9	11.7 ± 4.6		0.13
Medication	17.71%	13.76%		0.7
Antidepressant	3.13%	5.29%		0.2
Anticonvulsant	1.4%	1.6%		0.8
Analgesic	3.13%	1.6%		0.5
Vitamins	5.21%	2.12%		0.4
Combination therapy	5.21%	4.24%		0.8

**Table 2:** Comparison of the fission illusion conditions between patients with fibromyalgia and healthy controls. Data are plotted in the Figure 1(a).

Fission illusion	Fibromyalgia *n* = 41	Healthy controls *n* = 41	*p*
1F0B	1.07 ± 0.1	1.14 ± 0.2	0.11
1F1B	1.05 ± 0.09	1.1 ± 0.17	0.02
1F2B	1.58 ± 0.32	1.93 ± 0.0.34	<0.0001
1F3B	1.81 ± 0.38	2.27 ± 0.49	<0.0001
1F4B	1.98 ± 0.5	2.42 ± 0.67	0.001

**Table 3:** Comparison of the fission illusion conditions in patients with fibromyalgia with and without migraine. Data are plotted in the Figure 1(b).

Fission illusion	Fibromyalgia *n* = 24	Fibromyalgia with migraine *n* = 17	*p*
1F0B	1.06 ± 0.15	1.09 ± 0.14	0.51
1F1B	1.03 ± 0.07	1.06 ± 0.11	0.27
1F2B	1.62 ± 0.33	1.53 ± 0.31	0.39
1F3B	1.86 ± 0.44	1.76 ± 0.29	0.4
1F4B	2.01 ± 0.53	1.93 ± 0.49	0.6

**Table 4:** Comparison between 0B and 1B trials showing fusion illusory effect in all participants. Data are plotted in the Figure 1(c).

Flash	0 beep	1 beep	*p*
2	1.95 ± 0.45	1.77 ± 0.42	<0.0001
3	2.56 ± 0.5	2.48 ± 0.5	0.02
4	3.01 ± 0.52	2.76 ± 0.63	<0.0001

**Table 5:** Comparison of the fusion illusion conditions in patients with fibromyalgia and healthy controls. Data are plotted in the Figure 1(d).

Fusion illusion	Fibromyalgia *n* = 41	Healthy controls *n* = 24	*p*
2F0B	1.88 ± 0.39	2.08 ± 0.5	0.08
2F1B	1.71 ± 0.42	1.88 ± 0.42	0.7
3F0B	2.47 ± 0.46	2.73 ± 0.5	0.04
3F1B	2.43 ± 0.5	2.57 ± 0.5	0.3
4F0B	2.92 ± 0.53	3.19 ± 0.46	0.04
4F1B	2.8 ± 0.56	2.69 ± 0.76	0.49

## Data Availability

The data used to support the findings of this study are available from the corresponding author upon request from any qualified investigator for the purposes of replicating procedures and results.
